# A diagnostic pitfall of a sternal mass

**DOI:** 10.1002/ccr3.4418

**Published:** 2021-07-21

**Authors:** Walid Abid, Abdessalem Hentati, Ghassen Ben Halima, Mohamed Rebai, Ahmed Lasmar, Naourez Gouiaa, Imed Frikha

**Affiliations:** ^1^ Cardiovascular and Thoracic Department Habib Bourguiba Hospital University of Sfax Sfax Tunisia; ^2^ Laboratory of Pathological Anatomy and Cytology Habib Bourguiba Hospital University of Sfax Sfax Tunisia

**Keywords:** lymphoma, mass, primary, sternum, surgery

## Abstract

Primary sternal lymphoma represents a rare entity which must be evoked in front of a sternal mass especially as its treatment is different from that of sarcomas, the principal etiology of sternal masses.

## Graphical abstract

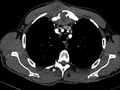

## 1 INTRODUCTION

Chondrosarcoma represents the most common type of malignant lesions of the sternum.^1^ Primary bone lymphoma (PBL) is a rare localization of invasion by Non‐Hodgkin lymphoma cells. The most common histological type is represented by the diffuse large B cells lymphoma (DLBCL). The sternum is an exceptional localization for PB‐DLBCL, accounting less than 1% of cases.^2^

We represent the case of a primary sternal DLBCL mimicking a sarcoma with difficult assessment.

## 2 THE CASE

**FIGURE 1:**
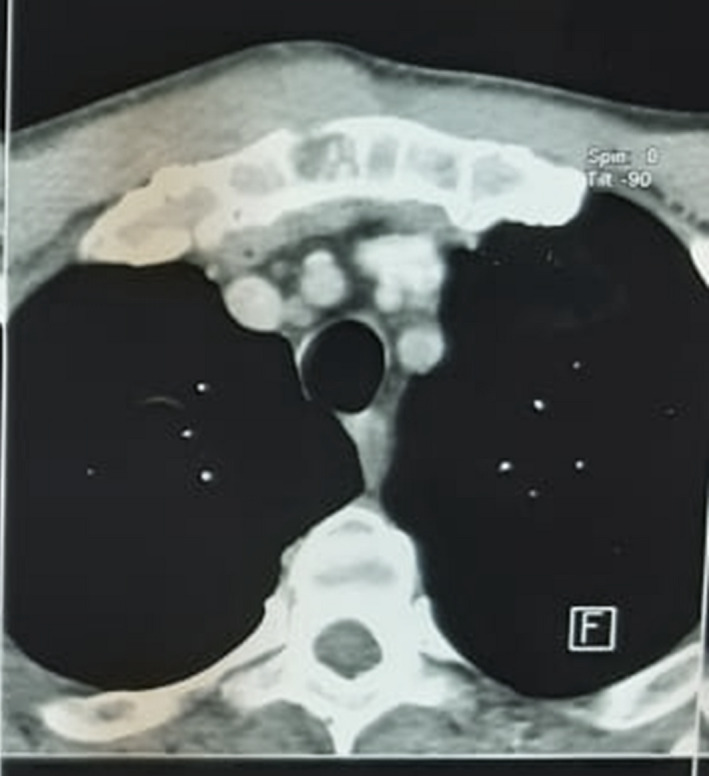
First CT of the chest

**FIGURE 2:**
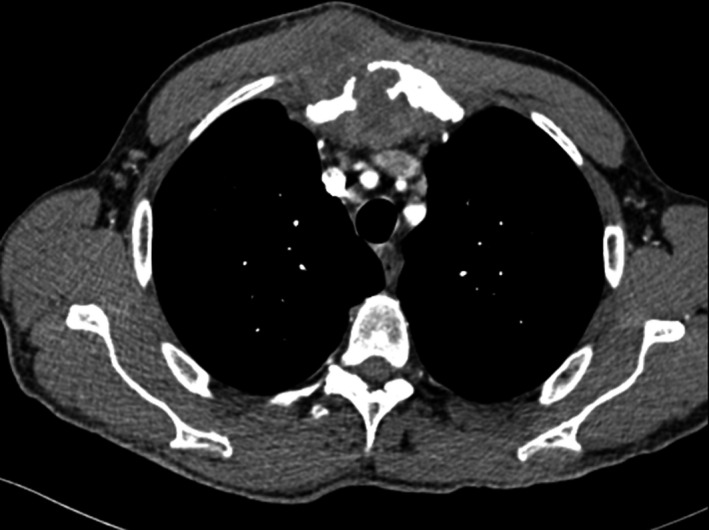
Second CT of the chest showing local progression

**FIGURE 3:**
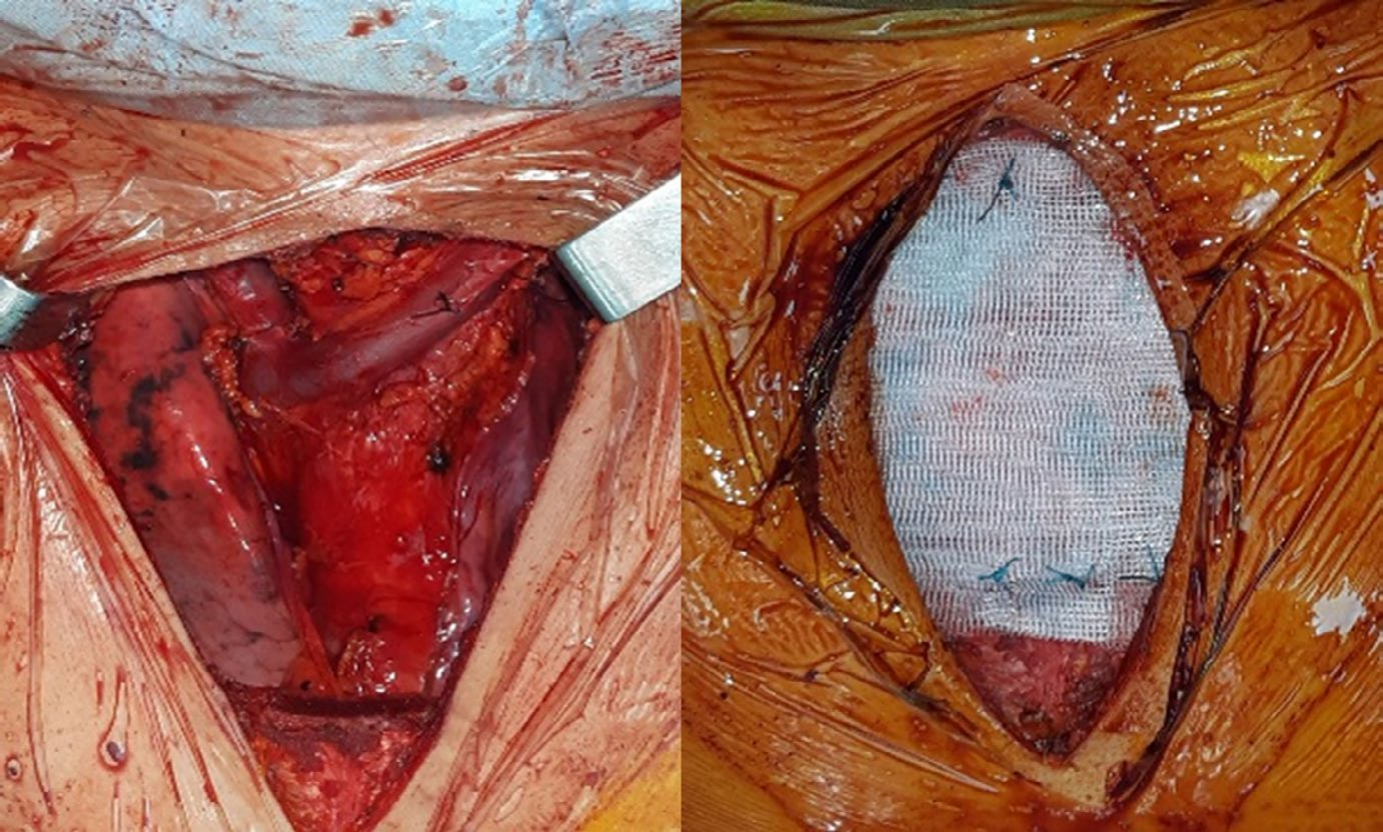
Per operative view: Resection and reconstruction

A 59‐year‐old man, heavy smoker, with no medical history, consulted for a sternal swelling starting from July 2019. The patient firstly consulted in November 2019. Systemic symptoms as fever, night sweating, or weight loss were absent. The clinical examination revealed a firm painful mass of the manubrium. There were no inflammatory signs of the skin. A first chest computed tomography (CT) revealed a poorly limited osteolytic lesion of the manubrium measuring 20 × 13 × 40 mm with multifocal interruption of the anterior and posterior bony cortex. There was a deep extension to the mediastinal tissue as well as to superficial layers (Figure 1). This scannographic aspect was very evocative of a chondrosarcoma. Computed tomographic scans of the head, abdomen, and pelvis did not reveal any suspicious lesion. There was no biological inflammatory syndrome, and LDH rate was normal. Tuberculosis tests were negative, excluding a tuberculosis destruction of the sternum. A needle biopsy was realized and concluded to a nonspecific inflammatory remodeling of striated muscle tissue. Unfortunately, it was not possible to continue the follow‐up because of COVID‐19 epidemic, and the patient was lost of sight for 3 months. Meanwhile, the sternal mass increased in size and become more painful. A second CT scan showed a progression of the tumor (48 × 40 × 58 mm) with a near contact with the brachiocephalic vein truncus (Figure 2). After multi‐disciplinary team discussion, and taking into consideration the high suspicion of a progressing sarcoma, it was decided to perform a surgical resection of the mass. Via a median skin incision facing the sternum, resection considered the whole manubrium with an extension to the upper part of the body of the sternum, the interior part of the clavicles, and the anterior arch of the two first ribs from both sides. In the anterior mediastinum, there was a tight adhesion to the thymus and so resection was enlarged to this later. Reconstruction was performed with a polytetrafluoroethylene (PTFE) mesh and covered by the two pectoralis major (Figure 3).

**FIGURE 4:**
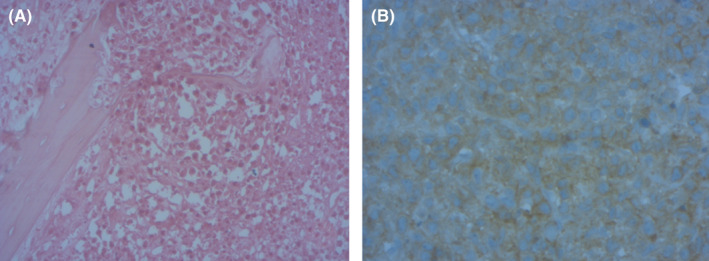
Bone dissociated by sheet of larges round cells (HE X200)

Postoperative course was uneventful. On macroscopy, manubrium was enlarged and the cut surface was white, homogeneous. The cortical bone was deformed with focal rupture. The posterior tissue contained lymph nodes measuring 5 mm‐3 cm. Histological examination revealed sternal and lymph nodes infiltration by sheets of large round cells with scanty cytoplasm and round nuclei containing two or three nucleoli; mitoses were numerous. There was no chondroid differentiation. Immuno‐histochemical staining showed high and diffuse positivity for CD20 and CD79a (Figure 4). Margins were negative. Prospectively, no secondary location is found and sternal location is the unique site. Level of LDH was normal. Based on morphologic finding and clinical findings (a single skeletal site with regional lymph node involvement), a primary B large cell lymphoma of sternum was posed. This tumor was staged IIE, and the patient received six cycles of R‐CHOP protocol.

## 3 DISCUSSION

Primary tumors of the sternum are rare, accounting less than 1% of all primary bone tumors.^3^ Ala‐Kulju et al^4^ reported only six cases of primary sternal tumor in 20 years. Most of these tumors are malignant, and the incidence of metastatic lesions, mainly from thyroid, kidney, lung, breast, or prostate gland cancers, is equal to primary ones.^4^ Despite of predominance of chondrosarcomas, other tumor may be presented as a bone mass, like lymphomas. Primary bone lymphoma (PBL) is infrequent and represents 5%‐7% of primary bone tumors, and 4%‐5% of extranodal non‐Hodgkin lymphomas (NHLs).^1^, ^2^ Extremities are the most common sites of PBL (femur 27%, pelvis 15%, tibia and fibula 13%), and so sternal localization is very rare.^1^

The absence of specific signs of sternal lymphomas makes the diagnostic difficult. In fact, clinical signs are usually a firm sternal painful swelling, progressing since several weeks.^3^ General symptoms like night sweating and loss of weight can be absent.^1^ Radiological explorations often show a lytic mass with effraction of the bonny cortex.^5^ Extension can be found to both deep and superficial layers, and lymph node involvement is less frequent in bone non‐Hodgkin lymphomas.^5^ The local extension is better explored by an MRI than by a scanner,^6^ and comparing to the rest of radiological tools, positron emission tomography coupled with computed tomography is more sensible in the assessment of extension, especially in multifocal forms, and helps in the follow‐up after treatment.^1^ All these elements explain why the diagnostic represents a challenge on itself.

Taking into consideration the frequency of malignant nature of sternal tumors, a histopathological confirmation is often necessary before any treatment, especially that in some cases, sternal resection with a curative oncological intent may be large with a complex reparation.^2^ Needle biopsy is considered as insufficient to make the diagnosis, and a surgical biopsy is often needed.^4^ In our case, and because of the special conditions with the COVID‐19 epidemic and the rapid progression of the mass, we did not perform a surgical biopsy, and we erroneously considered the sternal mass as a chondrosarcoma. Indeed, there is no radiological signs that are specific to a particular type of tumor. Furthermore, malignant degeneration of untreated benign tumors was reported, justifying an aggressive approach in some doubtful cases.^3^ In the case reported by Faries et al^5^, surgical resection was maintained even after an open biopsy with frozen examination in an antalgic intent and to reduce the size of the tumoral process.

The diagnostic of DLBCL is based on histological examination with immunohistochemical testing. Referred to World Health Organization classification of soft tissue and bone tumors, the group of a single skeletal site with or without regional lymph node involvement is considered primary lymphoma of bone. Expression of CD20 represents a therapeutic target. Indeed, anthracycline‐containing chemotherapy coupled to Rituximab is considered currently as the first‐line treatment of CD20‐positive DLBCLs.^1^ Radiotherapy at a dose of 30 Gy may be indicated after chemotherapy for the treatment of non‐Hodgkin lymphomas including extranodal sites, as it was revealed on a British randomized trial. Higher doses may be reserved in cases of suboptimal response to chemotherapy.^1^ Surgical resection, actually, do not have a place in the treatment of DLBCLs instead of surgical biopsy.

## 4 CONCLUSION

The diagnosis and treatment of sternal tumors may represent a real challenge. Even if chondrosarcomas are the most common, rare tumors such as DLBCLs must be kept in mind, especially that treatment strategies are different.

## CONFLICT OF INTEREST

None declared.

## AUTHOR CONTRIBUTIONS

WA and AH: collected data, research, and manuscript writing. GBH, MR, and AL: collected data and research. NG: collected data and correction of the manuscript. IF: involved in correction of the manuscript.

## ETHICAL APPROVAL

Published with written consent of the patient.

## Data Availability

All data are available.
